# Elephant Herding Optimization for Energy-Based Localization

**DOI:** 10.3390/s18092849

**Published:** 2018-08-29

**Authors:** Sérgio D. Correia, Marko Beko, Luis A. da Silva Cruz, Slavisa Tomic

**Affiliations:** 1 Instituto de Telecomunicações, Pólo II da Univ. de Coimbra, 3030-290 Coimbra, Portugal; lcruz@deec.uc.pt; 2 Instituto Politécnico de Portalegre, Departamento de Tecnologia, 7300-555 Portalegre, Portugal; 3 COPELABS, Universidade Lusófona de Humanidades e Tecnologias, Campo Grande 376, 1749-024 Lisboa, Portugal; mbeko@uninova.pt (M.B.); slavisa.tomic@ulusofona.pt (S.T.); 4 CTS/UNINOVA, Campus da FCT/UNL, Monte de Caparica, 2829-516 Caparica, Portugal; 5 Dep. de Eng. Elect. e de Computadores, Universidade de Coimbra, Pólo II, 3030-290 Coimbra, Portugal; 6 ISR/IST, LARSyS, Universidade de Lisboa, Av. Rovisco Pais 1, 1049-001 Lisbon, Portugal

**Keywords:** nature inspired algorithms, swarm optimization, elephant search algorithm, energy-based localization, acoustic positioning, wireless sensor networks

## Abstract

This work addresses the energy-based source localization problem in wireless sensors networks. Instead of circumventing the maximum likelihood (ML) problem by applying convex relaxations and approximations, we approach it directly by the use of metaheuristics. To the best of our knowledge, this is the first time that metaheuristics are applied to this type of problem. More specifically, an elephant herding optimization (EHO) algorithm is applied. Through extensive simulations, the key parameters of the EHO algorithm are optimized such that they match the energy decay model between two sensor nodes. A detailed analysis of the computational complexity is presented, as well as a performance comparison between the proposed algorithm and existing non-metaheuristic ones. Simulation results show that the new approach significantly outperforms existing solutions in noisy environments, encouraging further improvement and testing of metaheuristic methods.

## 1. Introduction

Localization of a source in wireless sensors networks (WSNs) has been commonly used in several real life applications, such as explorations (deep water, outer space, underground), surveillance and monitoring. [1,2,3,4]. In general, source localization algorithms can be categorized as range-free and range-based [5,6,7,8]. The former ones consider only information about connectivity and usually require a training phase in which a database is constructed [9,10]. Although less demanding in terms of computational burden, accuracy obtained by range-free methods is generally lower than the accuracy attained by the latter methods [9,10,11,12,13]. Range-based methods make use of the received signal in order to estimate the distance between the source and the receiving sensor node [14]. The distance information can be extracted from different measurements of the received signal, such as time of arrival, or time difference of arrival and received signal strength [15,16,17,18,19]. Nowadays, these different measurements are commonly integrated together, or combined with angle of arrival observations in order to enhance the localization accuracy [20,21,22].

Recently, energy-based localization has gained much attention in the signal processing community [23,24,25,26,27,28,29]. This localization approach considers averaging energy information of received acoustic signal data samples [30]. Energy-based acoustic localization, when considered for targets such as moving objects, has the property of varying slowly with time, thus, the acoustic energy signal can be sampled at a much lower rate. Therefore, the energy consumption for data transmission on individual sensor nodes will be reduced and the demand of communication bandwidth over wireless channels will also be lower [31]. By modeling the energy decay of an acoustic signal, transmitted within a WSN with one or more sources, a non-convex optimization problem arises. To deal with the non-convexity, several methods have been proposed in the literature. In [26], a weighted direct least-squares method with correction (WDC) was presented. This method is submissive to a correction technique leading to further performance gains, but its performance is degraded in high noise environments as the second-order noise terms are ignored. Wang and Yang showed in [29] that the non-convex problem can be relaxed as a convex semidefinite programming (SDP). Similarly, Beko showed in [24] that the originally non-convex problem can be solved by applying second-order cone programming (SOCP) relaxations. Although the methods in [24,29] perform well, even in noisy environments, their main drawback is their high computational complexity which increases significantly with the size of the network.

All of the above algorithms are based on applying certain approximations or relaxations to the original problem, causing discrepancies between the obtained and true solutions. These disparities might be large in the case where the applied relaxations are not sufficiently tight, resulting in high estimation errors. In order to circumvent this issue, this work proposes an entirely different approach. Instead of approximating the original localization problem, we tackle it directly, by resorting to a nature-inspired method, called the elephant herding optimization algorithm (EHO). This method was initially proposed by Wang et al. [32] applied to several benchmark functions. Essentially, it is a swarm based metaheuristic search method for solving optimization problems. The algorithm emulates the herding behavior of elephants in group. In nature, elephants belonging to different clans live together under the leadership of a matriarch, and the male elephants will leave their family group when reaching adulthood.

EHO has been applied to several optimization benchmark problems [33] and real life applications showing promising results in finding optimal solutions [34,35]. To the best of our knowledge, this method has not been used to solve energy based localization problems. Hence, in this work, EHO is adjusted and applied for energy-based positioning. While the main idea is preserved, optimal parameter tunning is sought through extensive simulations in order to capture the energy decay of acoustic signals between two sensor nodes. In this way, higher convergence rates are achieved together with near-optimal solutions. Since EHO does not resort to any type of relaxations, but rather tackles the original localization problem directly, its performance is less vulnerable to noise, thus, EHO outperforms the state of the art methods in high-level noise environments.

The paper is organized as follows. In Section 2 the energy decay model is introduces and the localization problem is formulated. Section 3 describes in detail the proposed EHO algorithm and the tunning procedure of its key parameters. Section 4 provides a performance analysis based on complexity and simulation results, and Section 5 summarizes the main conclusions and offers possible directions for future work.

## 2. Problem Formulation

Consider a 2-dimensional sensor network (The extension to a 3-dimensional scenario is straightforward), composed of *N* sensor nodes and a source node. The true (unknown) location of the source is denoted by x and the true (known) location of the *i*th sensor by $s_{i}$, where i=1,…,N. Our goal is to determine the unknown location of the source by exploiting energy measurements acquired by sensors. To do so, this work considers the decay model of an acoustic signal [30,31,36]. Each sensor makes *M* noisy measurements within a time window $T=M/f_{s}$, where $f_{s}$ is the sampling frequency. Therefore, we consider here the average energy signature over the time window [t−T/2,t+T/2]. Thus, according to [30,31,36], the received signal at the *i*th sensor can be modeled as

(1) $$z_{i}\left(t\right)=\frac{\sqrt{g_{i}}\varphi \left(t-\tau_{i}\right)}{||x-s_{i}{\left|\right|}^{\beta }/2}+\omega_{i}\left(t\right),\;i=1,\ldots ,N$$

where φ(t) is the intensity of the source signal, measured at a given default distance, $\tau_{i}$ is the time delay due to propagation from the source to the *i*th sensor, $\omega_{i}\left(t\right)$ represents an additive error modeled as Gaussian noise with zero mean and variance $\sigma_{\omega_{i}}^{2}$, $\sqrt{g_{i}}$ is the gain of the sensor *i* and β is the path loss exponent that that captures the decay rate of the signal [36]. The value of β typically falls within the interval [2,4] [5] (2 in free space and 4 in adverse indoor environments). In this work we consider β=2, since we consider signal propagation in free space, without reflections or reverberations.

To obtain the energy observations at the *i*th sensor, we average the readings over *M* signal measurements according to (2), i.e.,

(2) $$y_{i}=\frac{1}{M}\sum_{m=0}^{M-1}z_{i}^{2}\left(t_{s}+\frac{m}{f_{s}}\right),$$

where $t_{s}$ is the starting time.

Thus, combining (1) and (2) yields

(3) $$y_{i}=\frac{g_{i}P}{||x-s_{i}{\left|\right|}^{\beta }}+\nu_{i},\;\mathrm{for}\;\;\;i=1,\ldots ,N$$

where *P* is the transmitted power [7], and $\nu_{i}$ represents the measurement noise. According to the central limit theorem, for sufficiently large *M* (M>>30), the energy measurement noise of the *i*th sensor $\nu_{i}$ approximately obeys a Gaussian distribution, i.e., $\nu_{i}∼N\left(\sigma_{\omega }^{2},2\sigma_{\omega }^{4}/M\right)$ where $\sigma_{\omega }^{2}$ is the background noise level. Consequently we can subtract the mean $\sigma_{\omega }^{2}$ from (3), and assume $\nu_{i}∼N\left(0,\sigma_{\nu_{i}}^{2}\right)$, where $\sigma_{\nu_{i}}^{2}=2\sigma_{\omega }^{4}/M$ [25].

By employing the observations in (3), the maximum likelihood (ML) estimator of x can be formulated as [37]

(4) $$\hat{x}=\underset{\begin{array}{c} x \end{array}}{\mathrm{argmin}}\sum_{i=1}^{N}\frac{1}{\sigma_{\nu }^{2}}{\left(y_{i}-\frac{g_{i}P}{||x-s_{i}{\left|\right|}^{2}}\right)}^{2}$$

**Figure 1:**
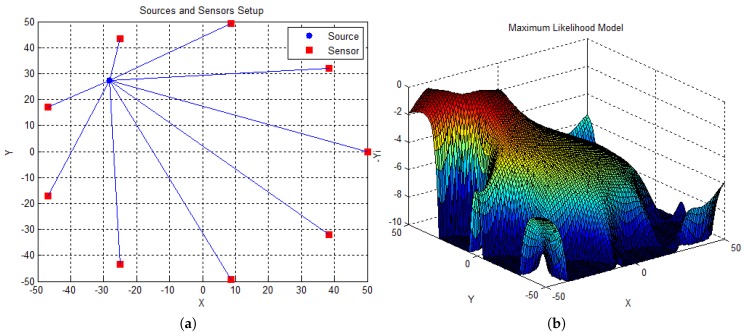
Graphic Representation of ML Model. (**a**) Sensors and Source Setup. (**b**) Surface representation.

The estimator in (4), is clearly non-convex. An illustration of a possible realization of (4) is given in Figure 1b. To plot the objective function in (4), a grid search was applied over the network topology shown in Figure 1a. Figure 1a depicts a WSN consisting of nine sensors and a source, deployed over a 100×100 m square region. The sensors are uniformly distributed on a circle, centered at [0,0]T, with the radius equal to half of the length of the region. The source is placed randomly in the considered space, and its true location was [−28, 28]. Other model parameters were set as $g_{i}=1\left(i=1,\ldots ,N\right)$, P=500. We added the measurements noise with $\sigma_{\nu_{i}}^{2}=-25$ dB.

With the goal of improving the visualization and interpretation of the objective function in (4), Figure 1b actually depicts the symmetric of (4). This means that we are now looking for a maximum, instead of a minimum of the function. From Figure 1b, it can be seen that the resulting surface is highly non-convex, comprising several local maxima and saddle points. Moreover, one can see that the global maximum is close to the true target’s position, i.e., at ${\left[XY,XY\right]}^{T}$. Since the singularities would imply a value of infinity at the sensor coordinates, a limit value of −10 for the graphical representation was considered. Due to the non-convexity of (4), finding its global minimum is an extremely difficult task. Therefore, in Section 3, we develop a sub-optimal estimator that provides accurate solution to the considered problem, especially in noisy environments.

## 3. Elephant Herding Optimization

### 3.1. Original EHO Algorithm

EHO algorithm was originally proposed by Wang et al. [32], and is essentially a swarm intelligence algorithm [38]. It is a metaheuristic search method which arises from modeling of herding behavior of elephants in nature. This particular behavior can be summarized as follows. The population of elephants contains a number of subgroups, known as clans, which comprise a number of elephants. Each clan moves under the leadership of a matriarch, while a number of male elephants that reached adulthood leave the clan they belong to and live in solitude. In terms of EHO, these behaviors can be modeled with two operators: clan update (which updates the elephants and matriarch current positions in each clan) and a separation (which enhances the population diversity at the later search phase) [32].

To be more specific, EHO is defined as follows. The entire elephant population is initially organized into *k* clans. After sorting elephants according to their fitness (corresponding to the evaluation of each elephant according to (4)), the clan updating operator is applied. Each member *j* of the *i*th clan moves according to the elephant matriarch, $c_{i}$, with the best fitness value, as

(5) $$x_{new,c_{i},j}=x_{c_{i},j}+\alpha \left(x_{best,c_{i}}-x_{c_{i},j}\right)r$$

where $x_{new,c_{i},j}$ and $x_{c_{i},j}$ are the new and old position of the *j*th elephant in the *i*th clan, respectively, α∈[0,1] is a tuning parameter that determines the influence of *i*th matriarch on $x_{new,c_{i},j}$, $x_{best,c_{i}}$ represents the fittest elephant individual in clan $c_{i}$, and r∼U[0,1] [32].

The position of the fittest elephant in the clan is updated according to

(6) $$x_{new,c_{i}}=\beta \;x_{center,c_{i}}$$

(7) $$x_{center,c_{i},d}=\frac{1}{n_{ci}}\sum_{j=1}^{n_{ci}}x_{c_{i},j,d}$$

where β∼U[0,1] is another tuning parameter which determines the influence of $x_{center,c_{i}}$ on $x_{new,c_{i}}$, *d* is a reference to the *d*th dimension, where 1≤d≤D and *D* being the dimension of the considered problem (in our case d=2, considering a two dimension problem), and $n_{ci}$ the number of elephants in the *i*th clan [32].

For the elephant with the worst fitness, the separating operator is applied in each interaction, moving the elephant to new positions, and replacing the elephant with the worst fitness in the *i*th clan. This is done as

(8) $$x_{worst,c_{i}}=x_{min}+\left(x_{max}-x_{min}+1\right)\;\psi ,$$

where $x_{max}$ and $x_{min}$ are respectively the upper and lower bound of the position of elephant individual, and ψ∼U[0,1] [32].

Therefore, the EHO algorithm implies iteratively applying (5)–(8) for a predefined number of iterations. Parameters such as the maximum number of iterations and population size are indirectly controlled by the number of clans and clan size, whereas α and β are considered fixed for a certain application.

The EHO algorithm was tested for several benchmark functions [32,39], and it showed promising results. Moreover, it was also considered in applications such as proportional–integral–derivative control tuning [34] and quality of web service composition [35].

### 3.2. Tunning of EHO Parameters

**Figure 2:**
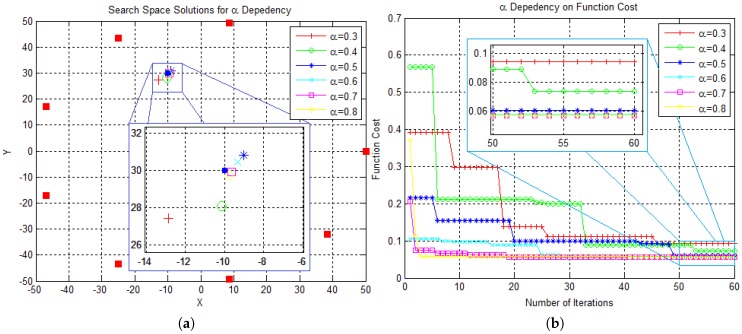
Parameter α Dependency Analysis. (**a**) Representation of the final solutions in search space; “•” denotes the true source location. (**b**) Convergence dependency on α in function cost.

In this section, the EHO algorithm is tested against various values of its key parameters in order to determine their influence on the convergence rate and the localization error, defined as the discrepancy between the true source position and the point fittest for fittest (4). Firstly, we consider the same setup used in Figure 1a, and vary α between 0.3 and 0.8 in order to analyze its influence on EHO algorithm. The results are presented in Figure 2.

Figure 2a plots the solutions of the proposed strategy for different values of α, whereas Figure 2b illustrates the dependency of the convergence rate on α. From Figure 2a, one can see that the solutions corresponding to higher α (e.g., α≥0.7) result in higher estimation accuracy, suggesting that these values of α are preferable for the problem at hand. Furthermore, Figure 2b shows that increasing α improves the convergence rate.

Since the EHO algorithm has a stochastic behavior due to (5) and (8), one specific scenario might not suffice to make any final conclusion, and Monte Carlo, $M_{c}$, runs should be considered. Hence, $M_{c}=10,000$ runs are performed and the root mean square error (RMSE) is considered as the performance metric. The RMSE is defined as

(9) $$RMSE=\sqrt{\sum_{i=1}^{M_{c}}\frac{||x-{\hat{x}}_{i}{\left|\right|}^{2}}{M_{c}}},$$

where ${\hat{x}}_{i}$ denotes the estimate of the true source location, x, in the *i*th Monte Carlo run.

**Figure 3:**
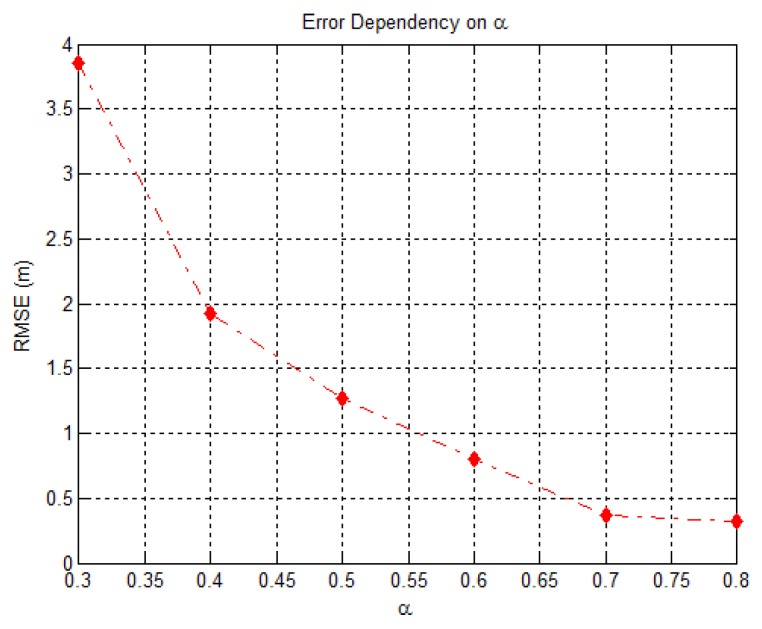
Root Mean Square Error (m) as a function of α, when $\sigma_{\nu }^{2}=-25$ dB and β=0.1.

Figure 3 illustrates the RMSE (m) versus α comparison, for the case where $\sigma_{\nu }^{2}=-25$ dB and β=0.1. The figure shows that as the value of α increases, the error decreases, which is in accordance with our previous indications. Therefore, since in EHO, α is the parameter that determines the influence of the matriarch and acts as a scale factor, higher values of α should be chosen. This implies higher dependency of $x_{c_{i},j}$ on $x_{new,c_{i},j}$.

**Figure 4:**
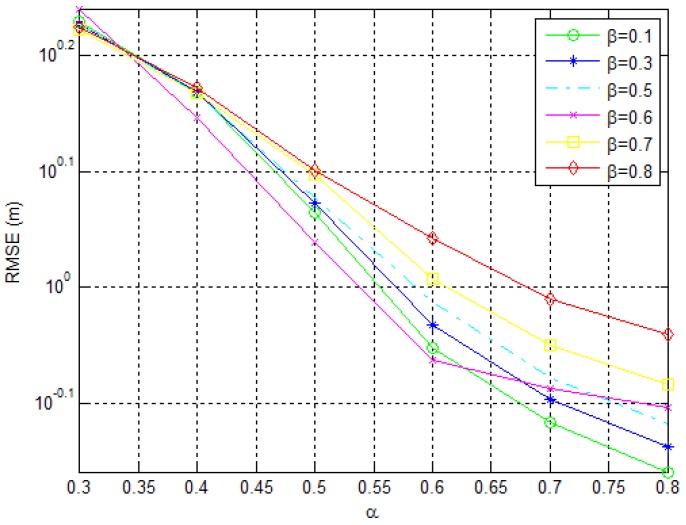
RMSE (m) dependency on α for different values of β.

The second object of our study is the parameter β, i.e., its influence on the RMSE, which is shown in Figure 4. It is worth remembering that this parameter defines the influence of $x_{center,c_{i}}$ on $x_{new,c_{i}}$. Low values of β will generate new points far from $x_{center,c_{i}}$ inducing high level of exploration, thus, we expect to see better accuracy of the algorithm for lower values of β in Figure 4. The results presented were generated by varying β from 0.1 to 0.8 under the same conditions as stated previously.

As we can see from Figure 4, although there is no significant variation of error when α is low (e.g., α≤0.4), in the case of preferable higher values of α, lower values of β should be used. Nevertheless, the effect of β on the localization accuracy is marginal.

The second set of parameters that are analyzed here concerns the population of elephants and its organization in clans. In EHO, the elephant population is organized in $c_{i}$ clans with $n_{ci}$ elephants per clan, hence, the population size is given by

(10) $$PopulationSize=n_{c_{i}}\;c_{i}.$$

**Figure 5:**
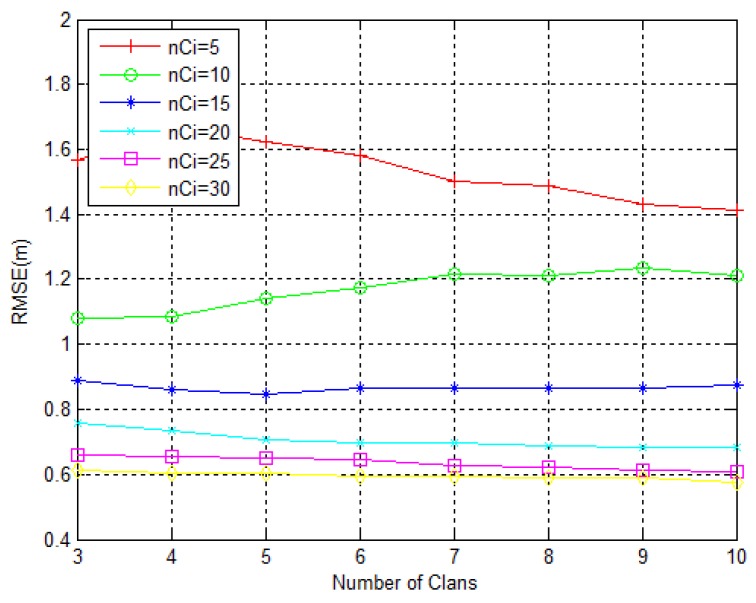
RMSE (m) dependency on the number of clans and $n_{c_{i}}$.

We consider the number of clans between three and 10 with an increment of five elephants, preserving previous simulation conditions regarding energy model, sensors’ layout and Monte Carlo runs. Notice that we are not considering a constant population size, since it will vary between 15 (three clans with five elephants) and 300 (10 clans with 30 elephants). The influence of these parameters on the RMSE is depicted in Figure 5.

As we can see from Figure 5, the increase of the number of elephants in each clan will produce a lower estimation error. However, for higher values of $n_{c_{i}}$ the decrease is much slower than for lower ones. This is because, with the increase of $n_{c_{i}}$, we are giving more importance to the exploitation of the regions of interest. Based on Figure 5 we see that for 15 (and more) elephants per clan is practically sufficient for the algorithm to converge, i.e., adding more clans does not do anything or does very little. The only way we can reduce the error in this case is to add more elephants in each clan.

**Figure 6:**
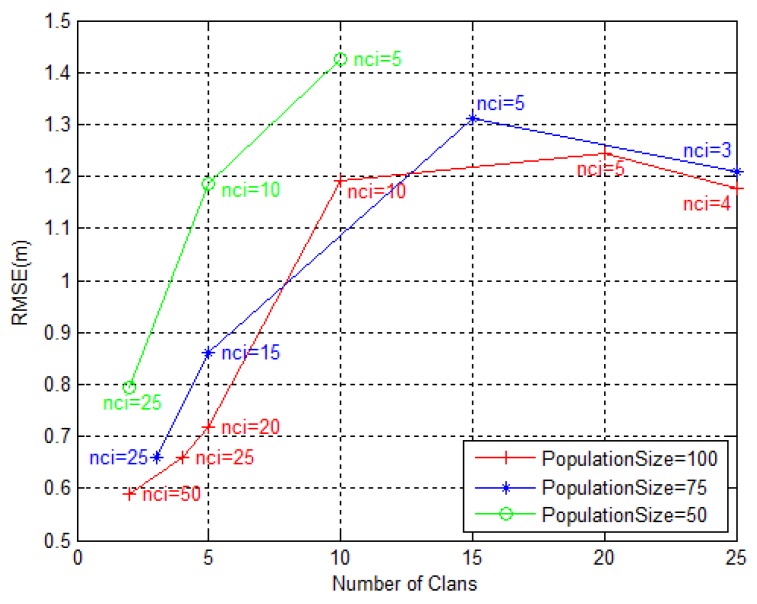
RMSE (m) dependency on the number of clans and $n_{c_{i}}$, for constant population size.

In the second stage, we considered a constant population size of 100, 75 and 50 elephants, and repeated the experiment under the same simulation conditions, but combining the number of clans and $n_{c_{i}}$ in order to keep the same population size. A summary of the setting is presented in Table 1, and the results are given in Figure 6.

From Figure 6, one can see that higher population sizes leads to lower RMSE in general, as anticipated. The three curves for different population size indicate that one should give more importance to the exploitation of the regions of interest, $n_{c_{i}}$, than to the exploration of the search space (number of clans). This could be explained to some extent by the intuition that the monitored region gets covered better by a lower number of herds comprising more elephants with greater degree of freedom, than vice versa.

## 4. Comparative Results

This section presents a set of results which offer insight to the reader about the performance of the considered localization algorithms. Both computational complexity and estimation accuracy of the algorithms are of interest, hence, the section is divided into its respective subsections.

### 4.1. Complexity Analysis

For a predefined maximum number of generations, EHO algorithm performs four operations: elephant sorting, update, separation and evaluation of the population. In the present work, elephant sorting was implemented by using the MATLAB^®^ sort function, which implements Quick Sort algorithm, of order O(nlog(n)). Update operator consists of two levels of cyclical operations, depending on the number of clans and the number of elephants in each clan, thus resulting in $O(n_{Clan}n_{c_{i}})$. The separating operator performs operations in each clan, rendering its complexity of $O(n_{Clan})$. Population evaluation concerns the total number of elephants, hence, the complexity of this operation is $O(n_{Clan}n_{c_{i}})$. Therefore, the complexity of solving the EHO algorithm is the junction of the four operations for a total of the maximum number of generations (MaxGen):

(11) $$O\left(MaxGen\left(n_{Clan}n_{c_{i}}log\left(n_{Clan}n_{c_{i}}\right)+n_{Clan}n_{c_{i}}+n_{Clan}+n_{Clan}n_{c_{i}}\right)\right)$$

where the four terms of the sum corresponds, respectively, to the four operations of the algorithm. Consequently, the complexity of solving the EHO algorithm is of order O(MaxGenN) where MaxGen is maximum generation of the population.

Given that *K* is the maximum number of steps on the bisection procedure in [40], Table 2 provides an overview of the considered algorithms together with their worst case computational complexities. From the table, it can be seen that the EHO algorithm has linear computational complexity with *N*, unlike most of the existing algorithms. This means that the execution time of EHO is likely to be lower than the execution time of the SDP, SOCP and WDC algorithms, which is a favorable property in most practical applications.

To empirically verify the complexity analysis, Table 2 also compares the average execution time of each algorithm implemented over 100 Monte Carlo runs. The network layout comprises nine sensors, the acoustic model was considered with P=500 and $g_{i}=1$, noise was added with $\sigma_{\nu_{i}}^{2}=-30$ dB. As expected from the complexity analysis, the table shows that the proposed algorithm is the second fastest, with average execution time well below 1 s.

### 4.2. Numerical Results

All algorithms considered in this section were implemented in MATLAB^®^ R2009b. The following experiments were performed on a platform consisting of a clustered computer with seven nodes, each with two Intel ©Xeon ©E5520 processors, 24 GB RAM, running Windows ©2008 Server HPC. For the proposed algorithm, we have considered EHO adjusted with its key parameters set according to the conclusions established in Section 3.2, i.e., α=0.7 and β=0.1. The number of clans were set 5 with $n_{c_{i}}=20$ elephants in each clan. A stopping criteria of 5000 function evaluation was used, implying a maximum of 50 generations (value of MaxGen considered in the complexity analysis) for a population size of 100 elephants. The performance of the proposed algorithm was compared with the SDP algorithm in [29], denoted by “SDP”, the bisection algorithm presented in [40], denoted in here by “EXACT”, the WDC algorithm in [26], and the SOCP algorithm in [24]. These algorithms are considered here as the state of the art of non-metaheuristic methods.

**Figure 7:**
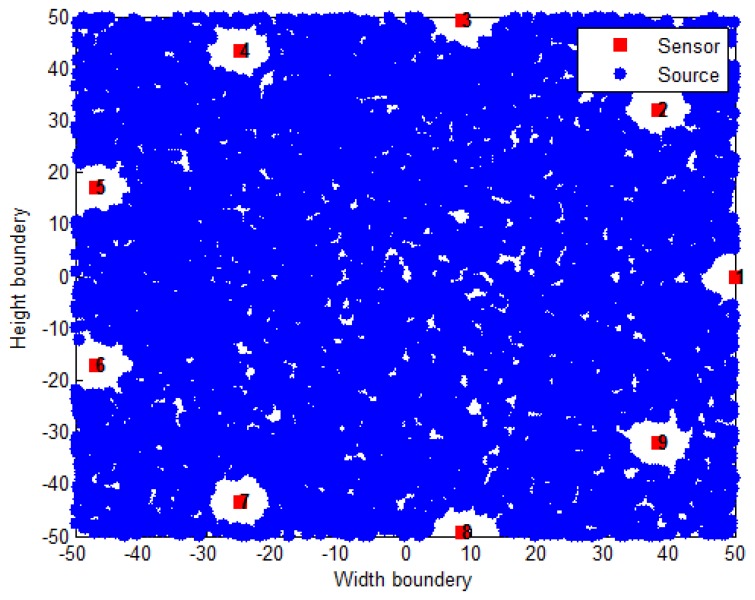
Random Distribution of 10,000 sources for N=9.

**Figure 8:**
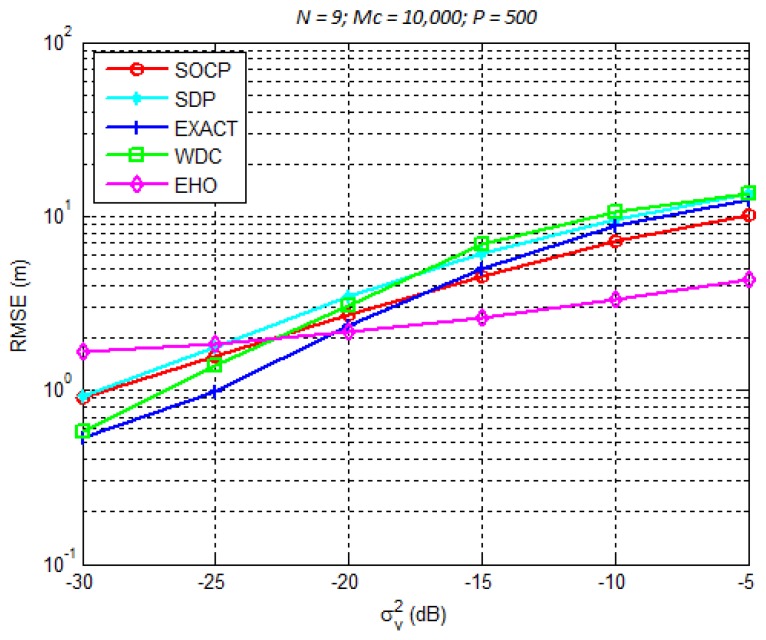
RMSE (m) versus $\sigma_{\nu_{i}}^{2}$ (dB) performance comparison for N=9.

**Figure 9:**
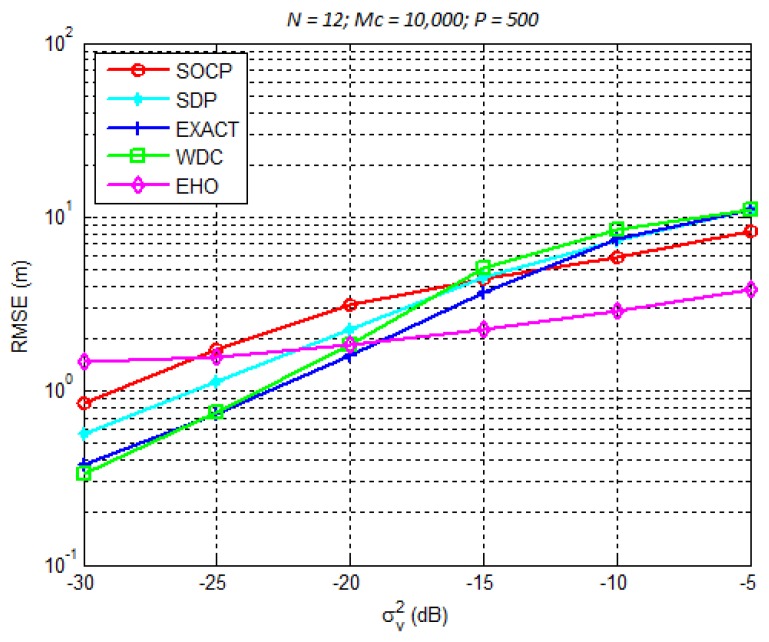
RMSE (m) versus $\sigma_{\nu_{i}}^{2}$ (dB) performance comparison for N=12.

The considered simulation setup included *N* sensors uniformly distributed on a circle whose radius was set to 50 m (where a minimum of three sensors is necessary since a 2-dimensional space is considered here), whereas the source was randomly distributed inside a $100\times 100\;m^{2}$ region (Figure 7). Note that, in all simulations presented here, MC = 10,000 are performed and (9) is used as the performance metric, in order to dissipate any effect of the source distribution in the search space, namely, sources located outside the sensors convex hull. First, we considered N=9 and studied the influence of the noise power, $\sigma_{\nu_{i}}$ on the estimation error. These results are plotted in Figure 8. Afterwards, N=12 was considered and the same study was performed. The results are presented in Figure 9.

**Figure 10:**
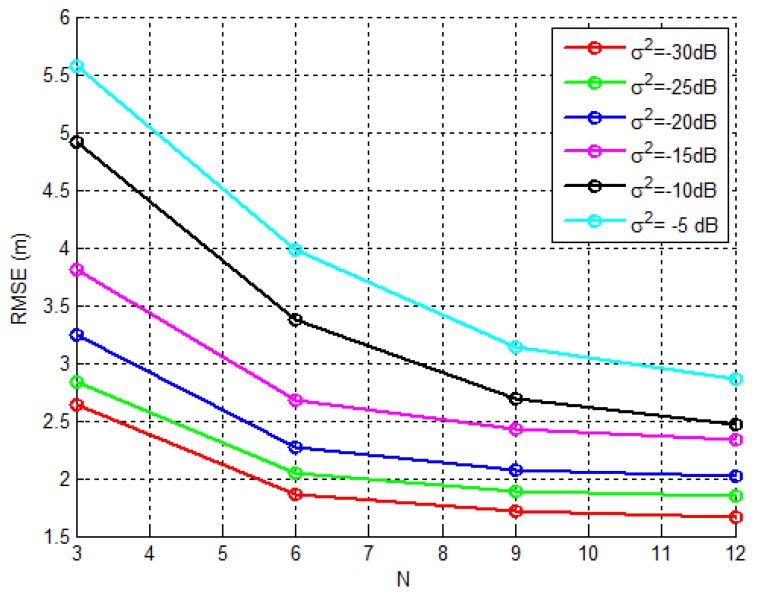
RMSE (m) versus N performance comparison for different $\sigma_{v}^{2}$.

From Figure 8 and Figure 9, we can make the following conclusions. There is an improvement of all considered algorithms when *N* is increased. Although marginally, the non-metaheuristic methods outperform the EHO algorithm for low $\sigma_{\nu_{i}}^{2}$. This result can be explained to some extent by the fact that these algorithms are derived based on the assumption that the noise power is small. Obviously, when this assumption holds, these methods perform well. However, when the noise power gets large, the EHO algorithm shows a significant gain in comparison with the non-metaheuristic methods. To illustrate this fact, we call the readers attention to the results shown in Figure 8 and Figure 9 for $\sigma_{\nu_{i}}^{2}=-5$ dB. For this setting, one can see that the gain achieved by EHO is more than 5 m for N=9, Figure 8, and more than 4 m for N=12, Figure 9. This is obviously a significant improvement in the estimation accuracy, which leads us to believe that metaheuristic methods have great potential in dealing with the problems of this sort. Lastly, it is important to study the influence of *N* on the localization error of the proposed algorithm, since for instance, some sensors might go off during the network lifetime. Therefore, Figure 10 illustrates RMSE versus *N* comparison, for different values of $\sigma_{v}^{2}$. Naturally, the figure shows improvement of the localization accuracy for any $\sigma_{v}^{2}$ when *N* is increased. Moreover, it can be seen from the figure that the proposed algorithm would work relatively well if any of the sensors goes off, as long as a minimum number of sensors is available (N=3 in the case of 2-dimensional’space).

## 5. Conclusions and Future Work

In this work, a new approach for the energy-based positioning problem was presented, based on nature-inspired EHO algorithm. In sharp contrast to the existing algorithms which apply approximations, relaxations to reach their final solution, EHO tackles the non-convex ML problem directly. By performing exhaustive simulations and analysis, the key parameters of the EHO algorithm were optimized to match the energy decay of acoustic signals, such that we could apply it to the problem of interest. The performance of the proposed algorithm was compared with the existing ones found in the literature. The simulation results showed that EHO significantly reduces the estimation error in all considered setups, especially in environments with high noise power. Furthermore, EHO represents an excellent trade off between the computational complexity and estimation accuracy, since it is significantly less complex than the optimization-based methods, and somewhat more complex than the linear ones. Finally, it is worth mentioning that the proposed algorithm works well in all considered scenarios as long as a minimum number of sensors is available, and benefits significantly from extra information when this number gets larger.

This work considered localization of a single, stationary, source node at a time. Generalizing the presented algorithm for simultaneous localization of multiple and non-stationary sources, possibly in cooperative WSNs, might be an interesting direction for future work. Similarly, integration of filters and Bayesian theory into EHO for its further improvement seems like an appealing and feasible idea. The present work has considered uniform sensor placement, where others networks architectures may be considered in future work, namely double, triple ring structures or random distribution. Finally, testing other nature-inspired algorithms deserves our further attention.

## Figures and Tables

**Table 1:** The considered simulation setting for constant population size experiment.

Pop.	Clan	$n_{\mathrm{ci}}$	Pop.	Clan	$n_{\mathrm{ci}}$	Pop.	Clan	$n_{\mathrm{ci}}$
100	2	50	50	2	25	75	3	25
	4	25		5	10		5	15
	5	20		10	5		15	5
	10	10					25	3
	20	5						
	25	4						

**Table 2:** Summary of the Considered Algorithms.

Algorithm	Description	Complexity	CPU Time (s)
WDC	The WDC algorithm in [26]	$O\left(N^{2}\right)$	1.74
SDP	The SDP algorithm in [29]	$O\left(N^{4.5}\right)$	3.5
SOCP	The SOCP algorithm in [24]	$O\left(N^{3.5}\right)$	2.6
EXACT	The bisection algorithm in [40]	$O\left(KN\right)$	0.03
EHO	The WLS algorithm in Section 3	$O\left(MaxGenN\right)$	0.23
